# Clinical Applications of Mesenchymal Stem/Stromal Cell Derived Extracellular Vesicles: Therapeutic Potential of an Acellular Product

**DOI:** 10.3390/diagnostics10120999

**Published:** 2020-11-24

**Authors:** Margherita Massa, Stefania Croce, Rita Campanelli, Carlotta Abbà, Elisa Lenta, Chiara Valsecchi, Maria Antonietta Avanzini

**Affiliations:** 1 Biochemistry, Biotechnology and Advanced Diagnostics Laboratory, Fondazione IRCCS Policlinico S. Matteo, 27100 Pavia, Italy; m.massa@smatteo.pv.it (M.M.); carlotta.abba@outlook.it (C.A.); 2 General Surgery Department, Fondazione IRCCS Policlinico S. Matteo, Department of Clinical, Surgical, Diagnostic & Pediatric Sciences, University of Pavia, 27100 Pavia, Italy; stefania.croce01@universitadipavia.it; 3 Center for the Study of Myelofibrosis, Biochemistry, Biotechnology and Advanced Diagnostics Laboratory, Fondazione IRCCS Policlinico S. Matteo, 27100 Pavia, Italy; r.campanelli@smatteo.pv.it; 4 Cell Factory, Pediatric Hematology Oncology, Fondazione IRCCS Policlinico S. Matteo, 27100 Pavia, Italy; elisa.lenta@yahoo.com; 5 Immunology and Transplantation Laboratory, Cell Factory, Pediatric Hematology Oncology, Fondazione IRCCS Policlinico S. Matteo, 27100 Pavia, Italy; c.valsecchi@smatteo.pv.it

**Keywords:** mesenchymal stem/stromal cells (MSCs), extracellular vesicles (EVs), medium/large sized enriched in microvesicles (m/lEVs), small EVs (sEVs), exosomes (Exos), microvesicles, secretoma, preclinical animal models

## Abstract

In the last decade, the secreting activity of mesenchymal stem/stromal cells (MSCs) has been widely investigated, due to its possible therapeutic role. In fact, MSCs release extracellular vesicles (EVs) containing relevant biomolecules such as mRNAs, microRNAs, bioactive lipids, and signaling receptors, able to restore physiological conditions where regenerative or anti-inflammatory actions are needed. An actual advantage would come from the therapeutic use of EVs with respect to MSCs, avoiding the possible immune rejection, the lung entrapment, improving the safety, and allowing the crossing of biological barriers. A number of concerns still have to be solved regarding the mechanisms determining the beneficial effect of MSC-EVs, the possible alteration of their properties as a consequence of the isolation/purification methods, and/or the best approach for a large-scale production for clinical use. Most of the preclinical studies have been successful, reporting for MSC-EVs a protecting role in acute kidney injury following ischemia reperfusion, a potent anti-inflammatory and anti-fibrotic effects by reducing disease associated inflammation and fibrosis in lung and liver, and the modulation of both innate and adaptive immune responses in graft versus host disease (GVHD) as well as autoimmune diseases. However, the translation of MSC-EVs to the clinical stage is still at the initial phase. Herein, we discuss the therapeutic potential of an acellular product such as MSC derived EVs (MSC-EVs) in acute and chronic pathologies.

## 1. Introduction

Extracellular vesicles (EVs), mediators of multiple biological functions, are released by almost all cells under physiological and/or pathological conditions, and when cellular activation or stress is present [1,2]. EVs, characterized by a phospholipid bilayer membrane, consist of particles with different size, defined as medium/large-sized EVs (m/lEV enriched in microvesicles) if ranging from 100 to 1000 nm or small EVs (sEV, enriched in exosomes) if ranging from 20 to 100 nm [3]. MVs are budded from the parental cell membrane and can contain microRNAs, mRNAs, proteins, and mitochondria. sEVs have endosomal biogenesis and can be composed of lipids, proteins, and nucleic acids [4]. We focus on clinical applications of EV derived from in vitro expanded Mesenchymal Stem/Stromal cells (MSCs), indicating that parental cells may have both cell-renewal (stem) and immunomodulatory capacities (stromal) [5,6]. Since the pioneer transplantations of MSCs have been documented [7,8], a huge number of studies have been reporting the beneficial effects of these cells in different clinical applications, due to either their ability to modulate the immune response or their capacity to differentiate into several lineages, being a relevant source to date for the therapy of pathologies characterized by inflammation and degenerative processes [9,10,11,12,13]. Moreover, MSC capability to trans-differentiate in endothelial cells and produce pro-angiogenic factors has allowed the investigation of their use in tissue regeneration (i.e., ischemic tissues) [14,15]. In recent years, the beneficial effects have been hampered by the possible, not to be excluded a priori, capacity that transplanted MSCs may unexpectedly differentiate or uncontrollably grow in the host [16,17], opening a scenario of still unknown complications for the receiver. These possible events lead the scientists to better investigate for in vivo cell free therapies. Initially, the therapeutic effects of MSCs were attributed to their capability to engraft in damaged tissues; however, it has been shown that only a small proportion of infused MSCs reach the targets [18] and several recent studies well documented how they exert their beneficial effects through a paracrine action by the release of EVs [19]. The MSC-EVs may reach distant sites taking advantage of extracellular fluids and mediate immune responses or tissue regeneration by different mechanisms of action, including the interaction with membrane specific receptors and/or the direct fusion of the EV membrane with target cell followed by the release of biological compounds in the cytosol [20,21].

The content of EVs is determined by parental MSCs that change their secretoma according with the inflammatory or noninflammatory characteristics of the local environment. This means that elevated concentrations of inflammatory mediators induce a noninflammatory response in these cells that release EVs able to suppress and limit the inflammation by blocking the action of M1 macrophages [22], the antigen presentation by dendritic cells [23], natural killer cells, and all the T cell subsets that may fuel the inflammatory function (i.e., Th1, Th17, and cytotoxic T cells) [24,25]. Conversely, MSCs in a noninflammatory microenvironment produce large quantities of inflammatory EVs aiming to induce and support the recruitment of cells able to mount and maintain an immune response [17]. Preclinical studies are showing that MSC-EVs have therapeutic effects quite comparable or even better than those obtained with transplanted MSCs [26,27]. MSC-EVs may significantly decrease the cell death in inflamed tissue by lowering the gene expression of pro-apoptotic Bax and increasing the expression of anti-apoptotic Bcl-2 [25]. Other cellular mechanisms involved in MSC-EVs inflammation control include the inhibition of the CD4^+^ T cells and CD8^+^ T cell proliferation by TGF-β action on IL-2, the tryptophan degradation to indoleamine 2,3-dioxygenase (IDO), molecule with immunosuppressive function, the modulation of the IL-1-IL-1R action by IL-1Rα, the induction of tolerogenic DCs, and the expansion of regulatory T cells (Tregs) [17,20,21,28,29]. At variance, it has recently been shown that the capacity to modulate the immune response may be differentially influenced by different MSC-EV preparations. Gouveia et al. [30] reported that MSC-EVs derived from both Bone Marrow (BM) and Adipose Tissue (AT) did not suppress in vitro lymphocyte proliferation, suggesting that cell–cell contact is not negligible to obtain the MSC immunosuppressive function. Similarly, Conforti et al. [31] reported a lower in vitro immunomodulatory effect of EVs on T and B cells, evaluated as proliferation and antibody secretion, as compared with their cellular counterpart. Moreover, it has been suggested that MSC-EVs efficacy could depend on both their biological content and, most importantly, on the recipient’s responsiveness towards MSC-EV [32]. Therefore, it could be crucial to define the functional activity of MSC-EV preparations and to test whether patients intended for treatment could be responsive to the envisioned MSC-EV therapy. The actual translation of MSC-EVs to the clinical stage still needs to be defined. In particular, several issues have to be investigated such as MSC-EV sources (adult or neonatal tissue), method of EV enrichment and characterization, and dosage and route of administration [3]. Nevertheless, MSC-EVs are candidates to become an actual alternative for clinicians in the near future. This may lead to a number of advantages such as the prevention of possible immune reaction against heterologous MSCs, or the formation of ectopic tissue and/or tumor masses, therefore improving the safety in a clinical setting. In addition, the number of curable pathologies would be increased by the fact that MSC-EVs are able to cross the blood brain barrier [33].

Below, we summarize the potential in vivo therapeutic applications of MSC-EVs taking into account their immunomodulatory and regenerative properties. Although we are aware that acellular product therapies are being tested in a huge number of pathologies, we analyze preclinical results from animal models of immune-mediated diseases (graft versus host disease) and organ injury (lung, kidney, skin), representing the large part of the registered clinical trials and also our field of interest. When available, we refer to results from clinical studies in humans. Closed or ongoing registered clinical trials are also reported.

## 2. EV Applications in Prevention or Treatment of Acute and Chronic Graft Versus Host Disease

Graft versus host disease (GVHD) is a frequent and severe consequence of allogeneic hematopoietic stem cell transplantation. It is an immunologically mediated process due to activated donor T cell proliferation homing toward target tissues and causing host tissue damage. About 50% of patients receiving immunosuppressant drugs may result in steroid-refractory [34]. Several reports underlined the beneficial MSC effects in these clinical contexts, while few data are available regarding the infusion of EVs, although it has been reported that MSC-EVs exert immunomodulatory effects as efficiently as MSCs [35].

Here, we review the reports of the in vivo and in vitro EV applications for GVHD prevention or therapy, starting from the first in vivo application in humans.

In 2014, Kordelas et al. [36] reported the safety and efficacy of MSC-EV infusions in a compassionate case of a patient with steroid-refractory acute GVHD (aGVHD), not eligible for MSC treatment. The EV preparations were analyzed for their content, and high quantities of the anti-inflammatory IL-10, TGF-β, and HLA-G were found. To reduce the risk of potential side effects, EVs were administered in 7 escalating doses over a period of 2 weeks. In vitro experiments demonstrated the reduction in inflammatory cytokine secretion by patient’s peripheral blood mononuclear cells (PBMCs) obtained at the end of EV infusions. In vivo downregulated activation of the patient’s PBMCs resulted in a decrease in serum pro-inflammatory cytokines and a significant improve of clinical symptoms.

The publication of this compassionate case was followed by a number of studies in animal models focused on either the prevention or cure of GVHD. EVs were used as an innovative approach in the prevention of aGVHD after allo-Hematopoietic Stem Cell Transplantation (HSCT) in a mouse model. In order to prevent life-threatening aGVHD, EVs were obtained from human umbilical cord-MSCs (UC-MSC-EVs) and administered intravenously. After infusions at day 0 and 7 after HSCT, the in vivo aGVHD development and the recipient survival were monitored. Recipients treated with human UC-MSC-EVs had significantly lower numbers of CD3^+^CD8^+^ cytotoxic T cells, and reduced serum levels of IL-2, TNF-α, and IFN-γ. At variance, a higher ratio of CD3^+^CD4^+^ and CD3^+^CD8^+^ T cells and higher serum levels of IL-10 were observed. These results were confirmed also by in vitro experiments, evaluating the immunosuppressive effects of human UC-MSC-EVs in mitogen-induced proliferation assay. This study indicated that the prophylactic effects of human UC-MSC-EVs for aGVHD was essentially due to proliferation suppression of allo-reactive T cells, altered imbalance of T cell subpopulations, inhibition of pro-inflammatory cytokine release, and induction of anti-inflammatory cytokines [37].

The immunologic aspect of recipients presenting aGVHD following human BM-MSC derived EVs infusion was evaluated in a mouse model described by Fujii et al. [38]. In this study, the expression of cytokines, chemokines, and growth factors as well as microRNAs (miRNAs) within the EVs was evaluated. miR-125a-3p was identified among other 10 miRNAs, as the most highly upregulated. They reported decreased frequency and number of both CD4^+^ and CD8^+^ effector T lymphocytes and an increased level of naïve T cells. Moreover, EV-treated aGVHD mice showed a higher number of Tregs compared to the nontreated group, leading to the conclusion that systemic infusions of human BM-MSC-EVs in aGVHD induced a decrease in effector and an increase in Treg cell subsets [38]. The beneficial therapeutic effect of MSC-EV infusions has been recently demonstrated in a xenogeneic model of GVHD, where an irradiated mouse was infused with human PBMCs. Immortalized human embryonic MSC-EVs were intraperitoneally injected beginning on day 1 after PBMC infusion. Subsequent doses were administered every 3 days until animal death or end of the study (day 34). The reduction in GVHD symptoms and the prolonged recipient survival were associated to the increased number of circulating Tregs, supporting the hypothesis that this represent a possible mechanism for EV immunomodulatory action. An interesting observation emerging from this study was that Treg induction by MSC exosomes (Exos) required activation of T cells by antigen presenting cells (APCs), demonstrated both in vitro and in vivo [39]. The therapeutic properties of EVs have been investigated in a chronic GVHD (cGVHD) mouse model [40]. Human BM-MSC-EVs were infused once a week for 6 weeks. Clinical signs and histological analysis were evaluated together with immunological aspects. EV treated mice showed a prolonged survival with amelioration of skin, lung, and liver fibrosis, compared to the nontreated group. In MSC-EV treated mice, activation of CD4^+^ T cells and their infiltration into the lung were reduced. The immunomodulatory effect of EVs was ascribed to the inhibition of Th17 and induction of Tregs. These observations were confirmed in vitro in co-culture experiments of BM-MSC-EVs with PBMCs obtained from patients with cGVHD [40]. The same authors recently described that BM-MSC-EV modulatory effects on other immune cells such as macrophages and B lymphocytes may have a role in preventing fibrosis in a mouse model of sclerodermatous cGVHD. In this study, a significant reduction in the infiltration of activated macrophages in the dermis of cGVHD mice was demonstrated after BM-MSC-EV treatment. Moreover, EV treatment was demonstrated to block the maturation of B cells and reduce the level of B-cell activating factor (BAFF), a key molecule for the survival, activation, development, selection, and differentiation of B cells [41].

**Figure 1:**
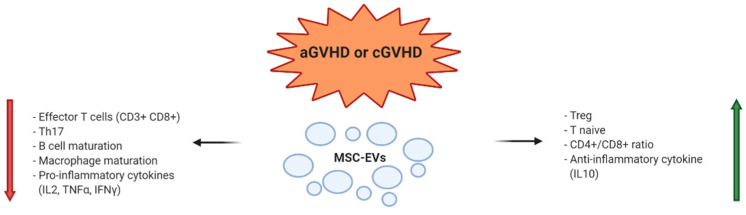
Mesenchymal stem/stromal cells-extracellular vesicle (MSC-EV) mechanisms of action in prevention and modulation of acute graft versus host disease (aGVHD) and chronic GVHD (cGVHD).

Summarizing the results of the considered preclinical applications in an immune mediated disease such as GVHD, we can underline that MSC-EVs, as their MSC counterpart, may act on almost all immune cells leading to a reduction in activated T lymphocytes and pro-inflammatory cytokines, together with the suppression of macrophage maturation and B cell response, and an induction of Treg cells and anti-inflammatory cytokines (Figure 1).

## 3. EV Applications in Acute and Chronic Kidney Disease

**Figure 2:**
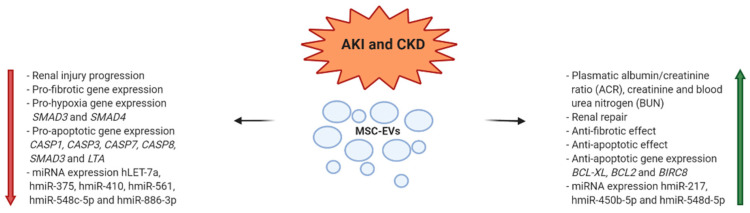
MSC-EV mechanisms of action in modulation of kidney diseases (h = human; m = murine).

A large percentage of patients with Acute Kidney Injury (AKI) or Chronic Kidney Disease (CKD) undergoes renal failure, requiring hemodialysis or even kidney transplantation [42,43]. Several preclinical studies are available regarding the use of EVs as a therapeutic approach [44]. In a number of studies, in vivo models describe the positive effect of EVs, ascribed to their regenerative tissue and immunoregulation capacities. These results have been obtained with heterogeneous experimental settings. EVs were derived from MSCs of different tissues, such as BM [45,46,47,48], cord blood [49], Warton’s Jelly [50,51], renal [52,53], liver [54,55], or urine [56]. Different doses and schedules were also applied [45,46,47,48,49,50,51,52,53,54,55,56]; the routes of administration included the tail infusion, the organ perfusion, or the direct administration in the kidney [45,46,47,48,49,50,51,52,53,54,55,56]. Different approaches in the induction of AKI and CKD animal models were also adopted [45,46,47,48,49,50,51,52,53,54,55,56]. We focus on preclinical approaches where human BM-MSCs were the source of EVs. In in vitro experiments, it was observed that BM-MSC-EVs stimulated the proliferation and conferred resistance of tubular epithelial cells to apoptosis; similar results were obtained in in vivo experiments where BM-MSC-EVs induced organ functional recovery in a Severe Combined Immunodeficiency (SCID) mouse model. The authors demonstrated, both in vitro and in vivo, that the biological effects were due to specific mRNA cargo associated with control of transcription, proliferation, and immunoregulation, transferred by the EVs to target tissues [57]. Gatti et al. [48] reported the beneficial effect of BM-MSC-EVs in favoring the recovery of AKI and CKD induced by ischemia-reperfusion injury. The EVs content was represented by mRNAs and miRNAs. Even in this study, the organ tissue repair was derived from inhibition of cellular apoptosis and stimulation of tubular epithelial cell proliferation. The same group investigated the effects of BM-MSC-EVs administered in single or multiple doses in an AKI SCID mouse model. Renal function was improved by single administration, but was normalized only after multiple injections. The protection was mainly ascribed to an anti-apoptotic effect of EVs, supported also by in vitro experiments where anti-apoptotic genes were upregulated in injured human tubular epithelial cells [47]. Lindoso et al. [58] investigated the role of MSC-EV cargo (miR-148b-3p, miR-410, miR495, and miR-548c-5p miR-886-3p) in the modulation of miRNAs inside renal proximal tubular epithelial cells in an in vitro model of ischemia-reperfusion injury induced by ATP depletion. Authors observed an enhanced incorporation of MSC-EVs in damaged tissues, with modulation of several miRNA expression involved in the repair and recovery process of epithelial cells. Many other preclinical studies are reported in literature supporting that the therapeutic effect of MSC-EVs in kidney diseases is due to the transfer of miRNAs, with activation of cell proliferation and survive of kidney tubular cells. [59,60,61,62] (Figure 2).

## 4. EV Applications in Lung Injury

**Figure 3:**
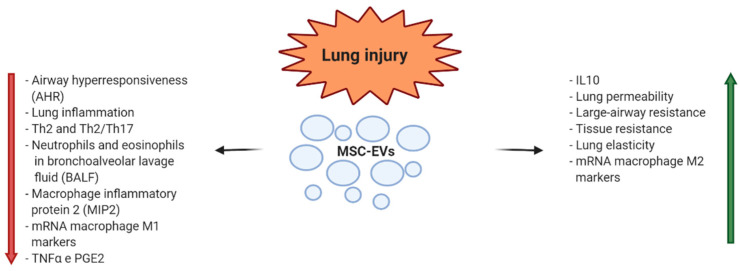
MSC-EV mechanisms of action in modulation of lung diseases.

Lung injury is characterized by severe airway inflammation with activation of alveolar epithelial cells, macrophages, pulmonary microvascular endothelial cells, and neutrophils causing tissue damages. The inflammatory response can be caused by inhalation of toxic particles or by infection. The consequences are acute or chronic pulmonary diseases, often with few clinical treatments available, characterized by difficulty in breathing and reduced pulmonary functions [63]. Due to their already mentioned capacities to modulate the immune response and to attenuate the tissue damages, observed in several preclinical studies [64,65], EVs have been proposed for clinical application in this context. In an endotoxin-induced mouse model of acute lung injury (ALI), the intratracheal administration of BM-MSC-EVs induced a decrease in inflammatory cytokines produced by neutrophils and macrophages. The result was a reduction in pulmonary edema and lung protein permeability [66]. Subsequently, the same authors demonstrated that intravenous MSC-EVs given 4 h after bacterial inoculation were effective in improving survival and attenuating ALI as their cellular counterpart. It was suggested that the shift of pro-inflammatory (M1) to the anti-inflammatory (M2) type of monocyte-macrophages was due to the transfer of COX2 mRNA from MSC-EVs to monocytes with a resultant increase in PGE2 secretion, responsible of the beneficial EV effects [67,68]. The anti-influenza and anti-inflammatory properties of MSC-EVs were also demonstrated in a preclinical large animal model of influenza virus that may cause acute respiratory disease in humans. The treatment with MSC-EVs in a pig model of influenza virus-induced ALI determined the reduction in virus shedding in the nasal swabs, influenza virus replication in the lungs, and virus-induced production of pro-inflammatory cytokines. The reduction in virus-induced lung lesions was evidenced by the histopathological findings [69]. Several data are consistent with therapeutic properties of MSC-EV early administration in experimental models of bronchopulmonary dysplasia (BPD) [70]. However, Willis et al. [71] demonstrated that even late infusion of EVs derived from Wharton’s Jelly-MSCs restored lung architecture and decreased pulmonary fibrosis and blood vessel loss in an experimental model of neonatal BPD. The interesting conclusion of the study was that EV infusions were effective not only to prevent the development of BPD but also to provide beneficial effects in established BPD. MSC-EVs were also tested in an animal model of allergic airway inflammation, where EVs resulted as effective as the parental MSCs in mitigating Th2/Th17-mediated allergic airway inflammation, with the reduction in pro-inflammatory cytokines in bronchoalveolar fluid [72]. Mechanical ventilation is the main supportive treatment of acute respiratory distress syndrome (ARDS), but it may lead to ventilator-induced lung injury (VILI). Recently, AT-MSC-EVs were administered by a small mechanical ventilator in a VILI mouse model. The study reported the protective effect on VILI of EV miR-146a in reducing the expression of pro-inflammatory cytokines by inhibiting Toll-Like Receptor 4 pathway, leading to the inhibition of calcium channel TRPV4 and extracellular calcium influx [73]. Recently, under compassionate use, an open-label trial for the treatment of 24 patients with SARS-Cov2 and ARDS was conducted using a commercial BM-MSC Exo preparation (ExoFlo^TM^, Direct Biologics). Amelioration of clinical symptoms was reported; however, a reinfusion was required after some days (3–4 days). The authors suggested that the presence of circulating proteases could inactivate Exos, requiring more than one dose to reach clinical results [74] (Figure 3).

## 5. EV Applications in Skin Wound Repair

Skin wound healing is characterized by four stages: (i) hemostasis, (ii) inflammation, (iii) proliferation, and (iv) maturation/remodeling [75,76]. For their capacity to control inflammation, activate angiogenesis, stimulate cell migration and proliferation, as well as to modulate the cellular differentiation, EVs can act in each of these phases [77].

In a severe burned skin rat model, it was demonstrated that human UC-MSC-EVs attenuated burn-induced inflammation. The infusion of EVs reduced the macrophage production of TNF-α and IL-1β and increased IL-10 levels. These observations were described as the result of the miR-181c transfer from EVs to macrophages, leading to the suppression of the Toll like receptor 4 signaling pathway and of the inflammatory response [78].

Ferreira et al. [79] showed in in vitro experiments that AT-MSC-EVs co-cultured with fibroblasts and keratinocytes enhanced cell proliferation, while the increase in cell migration was demonstrated by scratch wound healing assays. These key processes in skin wound healing are due to the activation of the AKT pathway, one of the major biochemical processes regulating migration of epithelial cells. Moreover, the EV effect on wound healing was evaluated in an excisional rat wound model, where topical application of MSC-EVs determined an acceleration of the process. All these data were confirmed in a subsequent study, where AT-MSC-EVs differentially expressed 292 out of 333 miRNAs (199 upregulated, 92 downregulated) able to inhibit genes NPM1, PDCD4, CCL5, and NUP62, and contributing to the regeneration of skin fibroblasts [80].

In an experimental rabbit model of skin wound, locally injected EVs obtained from BM- and AT-MSCs, induced tissue restoration even better than MSC treatment. Moreover, in this model, the reparative action of AT-EVs had significantly better results than that of BM-EVs [81].

A controlled release of entire secretoma from human AT-MSCs was obtained by a sponge-like alginate wound dressing and was presented as a possible application in a mouse model. In the treated group, skin appeared well regenerated, with a larger network of vascularization. The collagen deposition was increased as well as the number of mature fibroblasts; the epithelial layer, dermal papillae, cutaneous annexes, and connective matrix were also restored and were indicative of a good skin heal. An upregulation of specific wound-healing related proteins was also observed [82].

**Figure 4:**
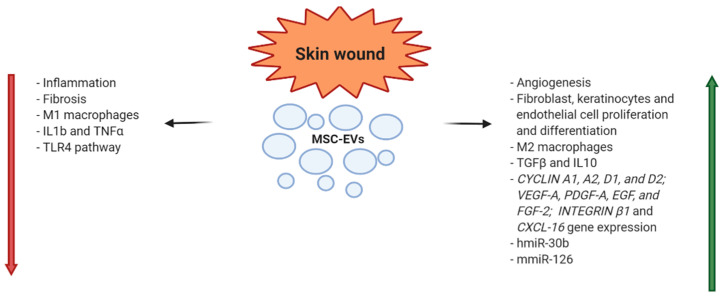
MSC-EV mechanisms of action in skin wound healing (h = human; m = murine).

In a recent preclinical study, it was demonstrated that human UC-MSC-EVs accelerated cutaneous wound healing by transferring miR-27b; in fact, the accelerated cutaneous wound healing observed in the EV treated group was abrogated by MSC transfection with miR-27b inhibitor [83] (Figure 4).

## 6. MSC-EV Possible New Tools in Advanced Clinical Approaches

### 6.1. Organ Pretreatment before Transplant

The difference existing between the need for transplants and organ availability still represents a major demand to be faced by the scientific community; new strategies are being investigated to ameliorate the balance worldwide. According with this need, in recent years, studies have been performed suggesting that the presence of MSC-EVs into the perfusion solution allows the amelioration of both function and viability of the organ to be transplanted [84,85,86]. The organ pretreatment with MSC-EVs has been demonstrated to reduce organ ischemia-reperfusion (IR) injury. The transfer of either proteins or genetic materials to the organ target cells through the MSC-EVs constitutes the rationale of this new approach. At present, the number of studies on MSC-EVs in the context of organ transplantation is limited; however, Gregorini et al. [87] applied this kind of procedure in a kidney donation after circulatory death (DCD) rat model. The tissue damage due to IR was limited by the use of MSC-EVs during hypothermic machine perfusion. Organs treated with MSC-EVs showed upregulation of genes encoding enzymes known to improve cell energy metabolism and genes encoding proteins involved in ion membrane transport. Interestingly, the addition of MSC-EVs during perfusion protects the kidney from ischemic injury by preserving the enzymatic machinery essential for cell viability [87]. The study by Stone et al. [88] investigated the same approach in a mice model of lung DCD. Decreased edema, neutrophil infiltration, and myeloperoxidase levels were observed in treated mice lungs, with a significant decrease in pro-inflammatory cytokines and upregulation of keratinocyte growth factor, prostaglandin E2, and IL-10 in the bronchoalveolar lavage. In general, the therapeutic benefits of MSC-EVs were ascribed to downregulation of immune cell activation as well as maintenance of endothelial barrier integrity.

### 6.2. Engineered MSC-EVs

EVs are natural transporters that may potentially reach a wide range of tissues following systemic administration, including the central nervous system as they have been reported to cross the blood–brain barrier [89]. They are characterized by a lipid membrane with an aqueous core and they may be loaded with an “extra content” of bioactive molecules or drugs. The loading of EVs is still a matter of debate since it could be achieved by a direct method, in which purified EVs are modified, or an indirect method in which parental cells are engineered [90,91]. The direct loading includes different procedures such as electroporation [92], sonication [93], and permeabilization [94] without altering the EV integrity. In the indirect method, the “extra content” is loaded into the parental cells from which EVs originate. Moreover, the delivery of the EV “extra content” may be driven to specific cells using genetically manipulated parental cells expressing “ad hoc” peptides on their membrane, therefore allowing the EV fusion with target tissues [92,95,96].

This approach may pave the way to a “personalized medicine” [97,98,99], as described by Pascucci et al. [100] who observed that paclitaxel-treated MSCs mediated strong anti-tumorigenic effects because of their capacity to take up the drug and later release it in EVs. In this study, paclitaxel-treated MSC-EVs induced a dose-dependent inhibition of human pancreatic adenocarcinoma cell proliferation as well as inhibition of tumor growth. In recent years, a number of papers have been published describing different methods performed to obtain a large amount of EVs charged with “extra content” [101] and new technological devices to make the delivery of EV “extra content” efficient and precise [102]. To improve and accelerate the utilization of these new tools in clinical applications, a number of criteria have been published to make the preparation of EVs more standardized and comparable among different laboratories [3,103]. However, the methods used to isolate and analyze EVs and their quality control still need to be clarified. Things are complicated by the heterogeneity of EVs, regarding both the dimensions and their composition [104,105]. In addition, the safety of EV preparations has to be defined by regulatory requirements [105] and meet good manufacturing practice standards [1,106] to allow their use in clinical settings.

## 7. Clinical Trials

The number of clinical trials registered on www.clinicaltrials.gov using MSC-EVs for therapeutic purposes has seen an increase in the last year (Table 1); however, few results are available.

The 2014 (NCT02138331) enrollment of 10 diabetes mellitus Type I patients. The aim was to evaluate the capability of UC-MSC-MVs to reduce the inflammatory state of the disease and improve the β-cell mass as well as the glycemic control. The state and results of this study are not available at present.

The 2016 (NCT not found) enrollment of 20 patients with chronic kidney disease. In this study, the patients received UC-MSC-EVs. Nassar et al. [107] reported a significant improvement of kidney functions, and the amelioration of the inflammatory immune reactions was observed.

The 2017 (NCT03437759) enrollment of 44 patients with macular degeneration. The study proposed to assess the safety and efficacy of MSC-Exos in promoting the healing of the damaged tissue. The study design also included the comparison of the therapeutic effect obtained with MSCs or MSC-EVs. The results are not available.

The 2019 (NCT03857841) enrollment of 18 preterm infants (<27 weeks of gestational age) at high risk for BPD. A phase I study, still enrolling patients, focused on the safety and efficacy of dose escalation intravenous infusion of BM-MSC-EVs (UNEX-42). In order to define the MSC-EV efficacy on the incidence and severity of BPD, the study will consider the duration of hospitalization, mechanical ventilation, supplemental oxygen therapy, and the use of postnatal steroids and of tracheal aspirate. The safety will be evaluated by physical examination, vital signs, adverse events, predefined complications of prematurity, clinical laboratory parameters, and chest x-rays.

The 2019 (NCT04134676) enrollment of 38 patients with chronic skin ulcer (completed). This study examined the therapeutic effect of human Wharton’s Jelly-MSC-EVs in wound healing. Results are not yet available.

The 2020 (NCT04356300) enrollment of 60 patients who will undergo surgical repair of acute type A aortic dissection immediately or presenting severe Multiple Organ Failure (not yet recruiting). In this study, the patients will be treated with human UC-MSC-EVs.

The 2020 (NCT04173650) enrolment of 10 patients with lesions due to Epidermolysis Bullosa (not yet started). Phase I/II, multi-center, randomized, vehicle controlled, study to assess the effectiveness and safety of topically applied allogeneic MSC-EVs from normal donor (AGLE-102).

The 2020 (NCT04223622) enrollment of 24 patients with osteoarthritis (not yet started). The study was designed to assess the therapeutic potential of AT-MSC secretome (either complete conditioned medium or EVs).

The 2020 (NCT04313647) enrollment of 27 healthy volunteers. The study will evaluate the safety and tolerance of aerosol inhalation containing escalating doses of Exos derived from allogenic AT-MSCs. The purpose of this study is to explore the safety and efficiency as well as provide a clinical dose reference for the subsequent trails of aerosol inhalation of MSC-Exos in the treatment of severe lung diseases (including severe lung infection, ARDS, and chronic obstructive pulmonary disease COPD).

The 2020 (NCT04276987) enrollment of 24 severe patients with novel coronavirus pneumonia. This was a pilot clinical trial registered by the same group (NCT04313647) to explore the safety and efficiency of aerosol inhalation of the Exos in the disease.

The 2020 (NCT04491240) enrollment of 90 severe patients hospitalized with novel SARS-Cov2 pneumonia. Following the clinical trial NCT04276987, to explore the safety and efficiency of the Exo preparation in the treatment of a larger population.

The 2020 (NCT04213248) enrollment of 27 patients with cGVHD with dry eye symptoms (still recruiting patients). The purpose of the study is to determine whether UC-MSC-Exos could alleviate the dry eye symptoms. The participants will receive artificial tears for 2 weeks to obtain the normalized baseline, followed by UC-MSC-Exos application for 2 weeks.

The 2020 (NCT04270006) enrollment of 10 patients with Periodontitis (still recruiting). The study will evaluate the regenerative effect of autologous AT-MSC-Exos injected locally into the periodontal pockets.

The 2020 (NCT04544215) enrollment of 60 patients with pulmonary infection caused by Gram-negative bacilli resistant to carbapenems (recruiting). The study will assess possible changes induced by aerosol inhalation of allogeneic AT-MSC-Exos in removing bacteria and in alveolar inflammation severity.

The 2020 (NCT04388982) enrollment of 9 patients with mild to moderate dementia due to Alzheimer’s Disease (not yet started). A phase I/II clinical trial designed to explore the safety and efficacy of the allogeneic AT-MSCs-Exos in the treatment of the disease.

## 8. Conclusions

Although at present most of the studies have been performed in preclinical animal models, the clinical trials are still ongoing, and MSC-EVs are considered by the scientific community an acellular biological product potentially interesting for therapies, with a number of advantages with respect to MSCs. MSC-EVs have a low immunogenicity, long half-life, in vivo stability, and a high efficacy of delivery because they are small and circulate readily, whereas most MSCs are trapped in the capillary bed of the lungs. In addition, their use avoids the transfer of cells which may have mutated or damaged DNA. Nevertheless, the therapeutic efficacy of MSCs has been shown to be related to their ability to actively respond to the microenvironment, while the infusion of purified EVs would represent a passive counterpart of MSCs. Understanding the molecular mechanism mediated by EV components will be an interesting goal for the future in order to define their therapeutic effect. MSC-EVs will certainly advance toward wide clinical testing since the International Society for Cellular and Gene Therapies (ISCT) and the International Society for Extracellular Vesicles (ISEV) recognize the potential of EVs from MSCs in clinical settings. However, there are still a number of critical parameters that need to be standardized, including production, purification, characterization, and storage of EVs. Additional important issues are the sources of EV originating cells, the total amount to be delivered, and the route of EV administration. The effort to address all these points will allow the evaluation of EV therapeutic efficacy comparable among both preclinical studies and appropriately powered clinical trials.

## Figures and Tables

**Table 1:** List of registered clinical trials from 2014 to 2020.

Clinical Trial Number	MSC Origin	Title	Year of Registration
NCT02138331	Cord blood	Effect of Microvesicles and Exosomes Therapy on β-cell Mass in Type I Diabetes Mellitus (T1DM)	2014
Not found	Umbilical cord	Chronic kidney injury	2016
NCT03437759	Human umbilical cord	MSC-Exos Promote Healing of Macular holes MHs	2017
NCT03857841	Bone marrow	A Safety Study of IV Stem Cell-derived Extracellular Vesicles (UNEX-42) in Preterm Neonates at High Risk for Bronchopulmonary Dysplasia	2019
NCT04173650	Bone marrow	MSC EVs in Dystrophic Epidermolysis Bullosa	2020
NCT04223622	Adipose tissue	Effects of ASC Secretome on Human Osteochondral Explants (ASC-OA)	2020
NCT04313647	Bone marrow	A Tolerance Clinical Study on Aerosol Inhalation of MSC-EXO in Healthy Volunteers	2020
NCT04276987	Adipose Tissue	A Pilot Clinical Study on Inhalation of MSC-EXO Treating Severe Novel Coronavirus Pneumonia	2020
NCT04491240	Adipose Tissue	Evaluation of Safety and Efficiency of Method of Exosome Inhalation in SARS-CoV-2 Associated PneumoniaSARS-Cov2 pneumonia	2020
NCT04213248	Human umbilical cord	Effect of UMSCs Derived Exosomes on Dry Eye in Patients With cGVHD	2020
NCT04270006	Adipose tissue	Evaluation of Adipose Derived Stem Cells Exo in Treatment of Periodontitis	2020
NCT04134676	Wharton’s jelly	Therapeutic Potential of Stem Cell Conditioned Medium on Chronic Ulcer Wounds	2020
NCT04356300	Bone marrow	MSC-EXO for Multiple Organ Dysfunction Syndrome After Surgical Repair of Acute Type A Aortic Dissection	2020
NCT04544215	Adipose tissue	A Clinical Study of MSC-EXO Nebulizer for the Treatment of Pulmonary Infection	2020
NCT04388982	Adipose tissue	Safety and Efficacy Evaluation of Allogenic Adipose MSC-Exos in Patients with Alzheimer’s Disease	2020

## Abbreviations

aGVHD	acute GVHD
AKI	Acute Kidney Injury
ALI	acute lung injury
APCs	antigen presenting cells
ARDS	acute respiratory distress syndrome
AT	Adipose Tissue
AT-MSCs	Adipose tissue-MSCs
BAFF	B-Cell Activating Factor
BM	Bone marrow
BM-MSCs	Bone marrow-MSCs
BPD	bronchopulmonary dysplasia
cGVHD	chronic GVHD
COPD	chronic obstructive pulmonary disease
CKD	Chronic Kidney Disease
DCD	donation after circulatory death
EVs	Extracellular Vesicles
Exos	exosomes
GVHD	Graft versus host disease
HSCT	Hematopoietic stem cell transplantation
IDO	Indoleamine 2,3-Dioxygenase
IR	ischemia-reperfusion
miRNAs	micro-RNAs
m/lEVs	medium/large sized enriched in microvesicles
MSC-EVs	MSC-derived extracellular vesicles
MSCs	Mesenchymal Stem/Stromal cell
MVs	microvesicles
PBMCs	peripheral blood mononuclear cells
SCID	Severe Combined Immunodeficiency
sEVs	small EVs
Treg	regulatory T cells
UC-MSC-EVs	umbilical cord-MSCs derived EVs
UC-MSCs	umbilical cord-MSCs
VILI	ventilator-induced lung injury
